# Supplementary material for: Comparison of Single Genome and Allele Frequency Data Reveals Discordant Demographic Histories
Source: G3 (Bethesda). 2017 Sep 11;7(11):3605–20. doi: 10.1534/g3.117.300259 (PMC5677151; doi:10.1534/g3.117.300259)
Supplement: Supplementary file 1 [file 3605FileS1.pdf]

Supplementary Materials

Supplementary Note 1: Individuals used in Schiffels and Durbin's (2014) MSMC Inference. Complete Genomics (Drmanac *et al.* 2010) sequences used by Schiffels and Durbin (2014) were statistically phased as described by the authors in their Online Methods. Parentheses designate which samples were used in the (((2), 4) and 8) haplotype analyses by Schiffels and Durbin (2014).

CEU: (((NA12891), NA12892), NA06985, NA06994)

CHB: (((NA18526, NA18537), NA18555, NA18558)

YRI: (((NA19238), NA19239), NA18501, NA18502)

Supplementary Note 2: Converting MSMC and SMC++ Models to *∂a∂i* and *ms* format

In their paper, Schiffels and Durbin (2014) presented their demographic models in terms of years and diploid population size (raw MSMC output had been rescaled by Schiffels and Durbin (2014) using the following parameters: generation time = 30 years/generation and mutation rate = 1.25×10^{-8} mutations per base per generation).

We converted their models into a format that could be used in ∂a∂i by scaling MSMC time-step intervals by 30 years/generation and in terms of $2N_{Ai}$ (the oldest ancestral size inferred by MSMC for each model) generations and scaling population sizes by N_{Ai}. To simulate these models in MaCS (Chen *et al.* 2009), we also generated ms-format versions of the models, with time points before present scaled by 30 years/generation and in terms of $4N_{Ai}$ generations and population sizes scaled by N_{Ai}. To confirm the validity of our step-wise models and to assess MSMC's ability to recapitulate its own demography, we ran MSMC 2-Haplotype on data simulated under the MSMC 2-Haplotype model, and found close concordance (**Figure S1**).

To confirm whether our method of converting MSMC demographic models to stepwise models was sound, and to assess MSMC's ability to recapitulate its own demography, we simulated 10 replicate 'genomes' for each population (CEU, CHB, YRI) in MaCS (Chen *et al.* 2009) under the stepwise MSMC 2-Haplotype model for each population (for additional simulation details, see **Methods**). MSMC 2-Haplotype was then run on these simulated data, and we found close correspondence between the MSMC original demographic models and those inferred by MSMC from the data simulated under the stepwise versions of the models (**Figure S1**). This indicates that the stepwise demographic models we generated from Schiffels and Durbin's (2014) MSMC results are an accurate representation of their models.

We converted Terhorst *et al.*’s (2017) SMC++ models to ∂a∂i and ms format in the same manner as the MSMC models. Their models were presented scaled by 29 years/generation and were inferred using a mutation rate of 1.25×10^{-8} mutations per base per generation.

Supplementary Note 3: Information on the 1000 Genomes Dataset

Unrelated 1000 Genomes Individuals (10 per population) used to generate the empirical 1000 Genomes SFS in this study.

CEU: NA06984, NA06985, NA06986, NA06989, NA06994, NA07000, NA07037, NA07051, NA07056, NA07347

CHB: NA18525, NA18526, NA18528, NA18530, NA18531, NA18532, NA18533, NA18534, NA18535, NA18536

YRI: NA18505, NA18517, NA18916, NA18923, NA18877, NA18909, NA18858, NA18865, NA19116, NA19096

Number of 100kb non-overlapping windows per population. Only sites that passed the 1000 Genomes “Strict Mask” filter were considered.

Population	Total number of 100kb windows	Total callable sites
**YRI**	26,435	1,949,360,368
**CEU**	26,411	1,949,349,189
**CHB**	26,403	1,949,340,620

Filters used to select 10kb putatively neutral windows from the 1000 Genomes dataset.

6333 x 10kb putatively neutral windows were selected with the Neutral Region Explorer (Arbiza *et al.* 2012).

Regions were selected that excluded:

1. Known genes
2. Gene bounds
3. Spliced ESTs
4. Segmental Duplications
5. CNVs
6. Self chain
7. Reduced Repeat Masker

Using the following parameters:

1. Miniumum region size: 200bp
2. Recombination rate (cM/Mb): 0.8
3. Genetic map: Decode
4. Human diversity: YRI; Individuals: All; Mask: Strict
5. Min BG selection coefficient: 0.95

Generating the 1000 Genomes SFS: We computed the folded SFSs following equation 1.2 of Wakeley's An Introduction to Coalescent Theory, reproduced below (Wakeley 2009):

$$\eta_i = \frac{\xi_i + \xi_{n-i}}{1 + \delta_{i,n-i}} \qquad 1 \leq i \leq [n/2]$$

where ξ_i represents the number of sites where the alternate allele is present at i copies, and $\delta_{i,n-i}$ is equal to 0 when $i \neq n - i$ and is equal to 1 when $i = n - i$. To obtain the proportional SFSs, we divided the number of sites in each bin (ie. singletons, doubletons, etc…) by the total number of segregating sites.

Supplementary Note 4: Assessing SFS fit.

Multinomial log-likelihoods were calculated for the proportional SFS expected under each demographic model relative to each of the three observed SFSs (Observed (Gutenkunst), 1000 Genomes (Whole Genome), and 1000 Genomes (Neutral)):

$$LogLikelihood(\Theta|x) = \sum_{i=1}^{n-1} x_i \, ln(p_i|\Theta),$$

where x_i is the number of SNPs in the observed SFS at count i in the sample, and p_i is the proportion of SNPs at frequency i under the demographic model Θ. Log-likelihoods were computed in this manner for each of the different demographic models. We also computed the log-likelihood for the observed data itself by replacing p_i with the observed proportion of SNPs at frequency i in the empirical data. The fit of different models was compared by examining their decrease in log-

likelihood compared to the fit of each of the observed SFSs to itself (**Table 1, S2, S5**). Multinomial log-likelihoods relative to the 1000 Genomes datasets were calculated with and without singletons (**Figure S7**).

Log-likelihoods were calculated for each SNP count absolute SFS using a Poisson likelihood relative to the Observed (Gutenkunst) SFS (**Table S3**) as in Lohmueller *et al.* (2010):

$$LogLikelihood(\Theta|x) = -\sum_{i=1}^{n-1}\theta(p_i|\Theta) + \sum_{i=1}^{n-1}x_i ln(\theta(p_i|\Theta)),$$

where $\boldsymbol{\theta}$ is the population scaled mutation rate (see **Methods** for calculation of theta for each model).

Supplementary Note 5: Scaling the MSMC models with an alternative mutation rate.

Schiffels and Durbin (2014) considered different scalings of their MSMC-inferred demographic models based on high and low mutation rates. To test whether the effect of differences in mutation rate between the studies may be responsible for discrepancies, in addition to generating the expected SFS for the MSMC models using the author's preferred scaling (μ = 1.25×10^{-8} mutations/bp/gen), we also rescaled the MSMC models using Gutenkunst *et al.*'s (2009) preferred higher mutation rate: $\mu = 2.35 \times 10^{-8}$ mutations/bp/gen (**Figure S3-S5**).

Differences in the mutation rate used to scale the MSMC models do not produce large qualitative differences for the MSMC models in the proportional SFS (**Figure S5**). However, some models provide a better fit when using the mutation rate from Gutenkunst *et al.* (2009). For instance, the rescaled CEU 8-Haplotype model is 78 log-likelihood units better (**Figure S5**, **Table S2**) using Gutenkunst *et al.*'s (2009) $\mu = 2.35 \times 10^{-8}$ mutations/bp/gen than when using Schiffels and Durbin's (2014) $\mu = 1.25 \times 10^{-8}$ mutations/bp/gen. Yet this improvement is not consistent across models or populations.

The conversion of the SFS from proportional to absolute used a value of θ calculated using the corresponding mutation rate of 2.35×10^{-8} mutations per base per generation (**Figure S6)**. The two different mutation rates again do not lead to major qualitative differences in MSMC model fit for the absolute SFS (**Figure S6; Table S3**). With the exception of the CHB 8-Haplotype model, all models rescaled to a mutation rate of 2.35×10^{-8} mutations/bp/gen fit worse than their 1.25×10^{-8} mutations/bp/gen counterparts (**Table S3**).

Supplementary Note 6: Impacts of Extreme Recent Growth and Neanderthal Admixture on MSMC Trajectories.

Extreme Recent Growth. In order to test the relative abilities of MSMC 2-Haplotype and 8-Haplotype to detect explosive recent growth, 10 independent replicates of 2-haplotype and 8-haplotype genomic (80 independent 30Mb chromosomes

per genome) datasets for CEU and YRI populations were simulated in MaCS under the original Gutenkunst model and the Gutenkunst model modified to include explosive recent growth as in (Tennessen *et al.* 2012) ("Gutenkunst + Growth"), with growth of 1.95% for CEU and 1.66% for YRI over the past 5115 years (Tennessen *et al.* 2012). MSMC 2-Haplotype and 8-Haplotype (with the recommended --fixedRecombination option for the 8-Haplotype inference) were then used to infer the demographic histories from the simulated datasets under each model (**Figure S8**).

Neanderthal Admixture: To test whether the addition of Neanderthal admixture might lead to MSMC trajectories that resemble those inferred by Schiffels and Durbin (2014), we simulated data under the Gutenkunst model with the addition of 2% Neanderthal mixture to the CHB/CEU populations 2000 generations ago from a Neanderthal population (Ne = 1000) that had been genetically isolated from humans for 16,000 generations after 44,000 generations of human/Neanderthal combined population size (N_A = 10,000) (Harris and Nielsen 2016). We simulated 50 replicates of 80 independent 30Mb chromosomes for each population (CEU, CHB, YRI) in MSMS (Ewing and Hermisson 2010) as MaCS was not able to model such a low amount of admixture for a small sample size (**Figure S9**). MSMC 2-Haplotype was then run on each replicate 'genome.'

Supplementary Note 7: Values of N_A tested when trying to improve the fit of the MSMC 2-Haplotype CEU model to the observed Gutenkunst SFS.

- N_A = 41,261: The oldest ancestral size inferred by MSMC (**Figure S10A-S13A**)
- N_A = 23,261: The population size inferred by MSMC at the time interval at which we trimmed the model (**Figure S10B-S13B**)
- N_A = 19,834: The harmonic mean of population sizes prior to the trimming cutoff (**Figure S10C-S13C**)
- N_A = 12,300: The population expansion size inferred by Gutenkunst *et al.* (2009) prior to the CHB+CEU/YRI split (**Figure 6, S10D-S13D**)
- N_A = 10,000: A commonly assumed ancestral human population size (Takahata *et al.* 1995; Adams and Hudson 2004) (**Figure S10E-S13E**)
- N_A = 7,300: The ancestral size inferred by Gutenkunst *et al.* (2009) (**Figure S10F-S13F**)

Table S1: Distribution of expected heterozygosity (π) in data simulated under each model for each population (CEU, CHB, YRI), compared to 1000 Genomes empirical distributions (Figure 2).

	Whole Genome, 100kb Windows								
	CEU			CHB			YRI		
**Model/Dataset**	**mean**	**median**	**sd[b]**	**mean**	**median**	**sd**	**mean**	**median**	**sd**
**1. 1000 Genomes (WG, 100kb)[a]**	**7.7E-04**	**7.3E-04**	**4.4E-04**	**7.2E-04**	**6.8E-04**	**4.5E-04**	**1.0E-03**	**9.8E-04**	**5.5E-04**
2. Gutenkunst	5.9E-04	5.8E-04	2.3E-04	5.6E-04	5.5E-04	2.3E-04	8.2E-04	8.1E-04	2.1E-04
3. MSMC 2-Hap	7.7E-04	7.5E-04	3.2E-04	7.5E-04	7.2E-04	3.4E-04	1.0E-03	1.0E-03	3.4E-04
4. MSMC 4-Hap	5.3E-03	5.4E-03	2.1E-03	5.0E-03	5.1E-03	2.0E-03	7.1E-03	7.1E-03	2.1E-03
5. MSMC 8-Hap	2.1E-04	2.0E-04	7.2E-05	2.6E-04	2.5E-04	9.5E-05	5.8E-03	5.8E-03	1.4E-03
6. SMC++	5.6E-04	5.6E-04	2.0E-04	5.2E-04	5.1E-04	2.0E-04	8.2E-04	8.1E-04	1.8E-04

	Whole Genome and Neutral, 10kb Windows								
	CEU			CHB			YRI		
**Model/Dataset**	**mean**	**median**	**sd**	**mean**	**median**	**sd**	**mean**	**median**	**sd**
**1a. 1000 Genomes (WG, 10kb)[c]**	**7.8E-04**	**6.7E-04**	**8.4E-04**	**7.3E-04**	**6.2E-04**	**7.9E-04**	**1.0E-03**	**9.1E-04**	**9.3E-04**
**1b. 1000 Genomes (Neut)[d]**	**9.4E-04**	**8.5E-04**	**5.1E-04**	**8.9E-04**	**8.0E-04**	**5.2E-04**	**1.2E-03**	**1.1E-03**	**5.4E-04**
2. Gutenkunst	6.0E-04	5.4E-04	3.3E-04	5.6E-04	5.0E-04	3.2E-04	8.2E-04	7.7E-04	3.3E-04
3. MSMC 2-Hap	7.8E-04	6.8E-04	5.4E-04	7.5E-04	6.5E-04	5.5E-04	1.0E-03	9.1E-04	5.5E-04
4. MSMC 4-Hap	5.3E-03	5.2E-03	3.3E-03	5.0E-03	4.8E-03	3.3E-03	7.1E-03	6.8E-03	3.6E-03
5. MSMC 8-Hap	2.0E-04	1.8E-04	1.2E-04	2.6E-04	2.3E-04	1.5E-04	5.8E-03	5.6E-03	2.3E-03
6. SMC++	5.6E-04	5.2E-04	3.2E-04	5.2E-04	4.7E-04	3.2E-04	8.2E-04	7.8E-04	3.1E-04

[a]Expected heterozygosity (π per site) calculated from non-overlapping 100kb windows from empirical 1000 Genomes whole genome data (10 individuals per population)
[b]Standard deviation
[c]Expected heterozygosity calculated from non-overlapping 10kb windows from empirical 1000 Genomes whole genome data (10 individuals per population)
[d]Expected heterozygosity calculated from non-overlapping 6333 x 10kb putatively neutral windows from empirical 1000 Genomes data (10 individuals per population)

Table S2. Multinomial log-likelihoods comparing the fit of various models to the observed SFS from Gutenkunst *et al.* (2009), including MSMC models rescaled using a higher mutation rate and data simulated under the Gutenkunst model and Gutenkunst model plus Neanderthal admixture.

CEU				
**Model**	**N_A (ancestral size)**	**Multinomial LL**	**Δ LL (Model - Data)**	**Figure**
Data to Data[a]	----	-21546	0	3A
Gutenkunst[b]	7,310	-21555	-9	3A
MSMC Simulated Data[d]	8,276	-21595	-49	7
SMC++[c]	9,960	-21599	-53	3A
MSMC Sim. Data (Gut. + Neanderthal)[d]	8,621	-21600	-54	S9
MSMC 2-Hap (μ 1.25)[e]	41,261	-21698	-152	3A
MSMC 8-Hap (μ 2.35) [e]	2,147	-21738	-192	3A
MSMC 2-Hap (μ 2.35) [e]	21,947	-21804	-258	3A
MSMC 8-Hap (μ 1.25) [e]	4,037	-21816	-270	3A
MSMC 4-Hap (μ 2.35) [e]	99,741	-22338	-792	3A
MSMC 4-Hap (μ 1.25)[e]	187,514	-22760	-1214	3A
**CHB**				
**Model**	**N_A (ancestral size)**	**Multinomial LL**	**Δ LL (Model - Data)**	**Figure**
Data to Data	----	-20154	0	3B
Gutenkunst	7,310	-20202	-48	3B
MSMC Sim. Data (Gut.)	8,701	-20276	-122	7
SMC++	10,096	-20277	-123	3B
MSMC Sim. Data (Gut + Neanderthal)	9,001	-20286	-132	S9
MSMC 8-Hap (μ 2.35)	3,014	-20339	-185	3B
MSMC 8-Hap (μ 1.25)	5,666	-20343	-188	3B
MSMC 2-Hap (μ 1.25)	41,126	-20370	-216	3B
MSMC 2-Hap (μ 2.35)	21,876	-20516	-362	3B
MSMC 4-Hap (μ 2.35)	101,723	-21053	-899	3B
MSMC 4-Hap (μ 1.25)	191,238	-21411	-1257	3B
**YRI**				
**Model**	**N_A (ancestral size)**	**Multinomial LL**	**Δ LL (Model - Data)**	**Figure**
Data to Data	----	-29630	0	3C
Gutenkunst	7,310	-29647	-17	3C
MSMC Sim. Data (Gut + Neanderthal)	7,059	-29653	-24	S9
MSMC Sim. Data (Gut.)	6,507	-29657	-27	7
SMC++	9,963	-29779	-150	3B
MSMC 2-Hap (μ 1.25)	41,100	-30003	-373	3C
MSMC 2-Hap (μ 2.35)	21,862	-30260	-630	3C
MSMC 8-Hap (μ 2.35)	70,222	-30662	-1033	3C
MSMC 8-Hap (μ 1.25)	132,017	-31282	-1652	3C
MSMC 4-Hap (μ 2.35)	109,492	-31930	-2301	3C
MSMC 4-Hap (μ 1.25)	205,845	-32976	-3346	3C

[a]Denotes the best log-likelihood possible when replacing the proportions predicted by the model with the observed proportions from the SFS used by Gutenkunst *et al.* (2009) in their inference (see **Supplementary Note 4**).
[b]Denotes the model inferred by Gutenkunst *et al.* (2009) fit to the observed SFS
[c]Denotes the model inferred by Terhorst *et al.* (2017) using SMC++, a combined approach
[d]Denotes the model based on demographic inference using MSMC on data simulated under the Gutenkunst model of demographic history, or under the Gutenkunst model plus Neanderthal admixture.
[e]Denotes the demographic models inferred by Schiffels and Durbin (2014) using MSMC on 2, 4 and 8 haplotypes and scaled using a mutation rate of 1.25×10^{-8} mutations/bp/gen ("μ 1.25") or 2.35×10^{-8} mutations/bp/gen ("μ 2.35")

Table S3. SNP count Poisson likelihoods comparing the fit of various models to the observed SFS from Gutenkunst *et al.* (2009), including MSMC models rescaled using a higher mutation rate and data simulated under the Gutenkunst model and Gutenkunst model plus Neanderthal admixture.

CEU						
**Model**	**N_A (ancestral size)**	**μ[f]**	**θ[g]**	**Poisson LL**	**Δ LL (Model - Data)**	**Figure**
Data to Data[a]	----	----	----	44688	0	4A
Gutenkunst[b]	7,310	2.35	2,776	44679	-9	4A
MSMC Sim. Data (Gut.)[c]	8,276	2.35	3,143	44633	-55	7
MSMC Sim. Data[c] (Gut + Neanderthal)	8,621	2.35	3,274	44632	-56	S9
SMC++[d]	9,960	1.25	2,012	44593	-86	4A
MSMC 2-Hap (μ 1.25)[e]	41,261	1.25	8,335	44410	-278	4A
MSMC 2-Hap (μ 2.35)[e]	21,947	2.35	8,335	43481	-1207	4A
MSMC 8-Hap (μ 1.25)[e]	4,037	1.25	815	41551	-3137	4A
MSMC 8-Hap (μ 2.35)[e]	2,147	2.35	815	41411	-3277	4A
MSMC 4-Hap (μ 1.25)[e]	187,514	1.25	37,878	8568	-36120	4A
MSMC 4-Hap (μ 2.35)[e]	99,741	2.35	37,878	-9066	-53754	4A
**CHB**						
**Model**	**N_A (ancestral size)**	**μ**	**θ**	**Poisson LL**	**Δ LL (Model - Data)**	**Figure**
Data to Data	----	----	----	41342	0	4B
Gutenkunst	7,310	2.35	2,776	41294	-49	4B
MSMC Sim. Data (Gut.)	8,701	2.35	3,304	41219	-123	7
MSMC Sim. Data (Gut + Neanderthal)	9,001	2.35	3,418	41211	-132	S9
SMC++	10,096	1.25	2,039	41166	-176	4B
MSMC 2-Hap (μ 1.25)	41,126	1.25	8,307	40965	-378	4B
MSMC 2-Hap (μ 2.35)	21,876	2.35	8,307	39873	-1469	4B
MSMC 8-Hap (μ 2.35)	3,014	2.35	1,145	39600	-1743	4B
MSMC 8-Hap (μ 1.25)	5,666	1.25	1,145	39578	-1764	4B
MSMC 4-Hap (μ 1.25)	191,238	1.25	38,630	5282	-36061	4B
MSMC 4-Hap (μ 2.35)	101,723	2.35	38,630	-13233	-54576	4B
**YRI**						
**Model**	**N_A (ancestral size)**	**μ**	**θ**	**Poisson LL**	**Δ LL (Model - Data)**	**Figure**
Data to Data	----	----	----	80240	0	4C
Gutenkunst	7,310	2.35	2,776	80223	-17	4C
MSMC Sim. Data (Gut + Neanderthal)	7,059	2.35	2,681	80216	-24	S9
MSMC Sim. Data (Gut.)	6,507	2.35	2,471	80213	-27	7
SMC++	9,963	1.25	2,012	80047	-193	4C
MSMC 2-Hap (μ 1.25)	41,100	1.25	8,302	79785	-455	4C
MSMC 2-Hap (μ 2.35)	21,862	2.35	8,302	79188	-1052	4C
MSMC 8-Hap (μ 1.25)	132,017	1.25	26,667	41002	-39238	4C
MSMC 8-Hap (μ 2.35)	70,222	2.35	26,667	35058	-45182	4C
MSMC 4-Hap (μ 1.25)	205,845	1.25	41,581	30801	-49439	4C
MSMC 4-Hap (μ 2.35)	109,492	2.35	41,581	15319	-64921	4C

[a]Denotes the best log-likelihood possible when replacing the counts predicted by the model with the observed counts (see **Supplementary Note 4**).

[b]Denotes the model inferred by Gutenkunst *et al.* (2009) fit to the observed SFS

[c]Denotes the model based on demographic inference using MSMC on data simulated under the Gutenkunst model of demographic history, or under the Gutenkunst model plus Neanderthal admixture.

[d]Denotes the model inferred by Terhorst *et al.* (2017) using SMC++, a combined approach

[e]Denotes the demographic models inferred by Schiffels and Durbin (2014) using MSMC on 2, 4 and 8 haplotypes and scaled using a mutation rate of 1.25×10^{-8} mutations/bp/gen (“μ 1.25”) or 2.35×10^{-8} mutations/bp/gen (“μ 2.35”)

[f]Mutation rate used for scaling models and calculating θ (units of 10^{-8} mutations per base per generation)

[g]$\theta = 4N_A\mu$ * sequence length (4.04Mb)

Table S4. Multinomial log-likelihoods comparing the fit of various models to observed SFSs generated from 1000 Genomes data from the whole genome or from 6333 x 10kb putatively neutral regions, with and without singletons.

	CEU: Figure 5A							
	Observed SFS: 1000 Genomes, Whole Genome				Observed SFS: 1000 Genomes, Neutral			
	SINGLETONS EXCLUDED		SINGLETONS INCLUDED		SINGLETONS EXCLUDED		SINGLETONS INCLUDED	
**Model**	**LL[e]**	**Δ LL[f]**	**LL**	**Δ LL**	**LL**	**Δ LL**	**LL**	**Δ LL**
Data to Data[a]	-8,220,905	0	-11,277,484	0	-265,585	0	-362,557	0
Gutenkunst[b]	-8,221,366	-461	-11,291,161	-13,677	-265,693	-108	-363,243	-686
SMC++[c]	-8,231,371	-10,466	-11,288,117	-10,633	-265,879	-294	-362,852	-295
MSMC 2-Hap[d]	-8,237,151	-16,245	-11,324,181	-46,697	-266,054	-469	-363,790	-1,233
MSMC 8-Hap[d]	-8,243,264	-22,359	-11,402,529	-125,045	-266,496	-911	-367,159	-4,602
MSMC 4-Hap[d]	-8,303,995	-83,090	-11,730,091	-452,606	-268,078	-2,493	-376,070	-13,514

	CHB: Figure 5B							
	Observed SFS: 1000 Genomes, Whole Genome				Observed SFS: 1000 Genomes, Neutral			
	SINGLETONS EXCLUDED		SINGLETONS INCLUDED		SINGLETONS EXCLUDED		SINGLETONS INCLUDED	
**Model**	**LL**	**Δ LL**	**LL**	**Δ LL**	**LL**	**Δ LL**	**LL**	**Δ LL**
Data to Data	-7,704,199	0	-10,441,302	0	-252,000	0	-337,894	0
Gutenkunst	-7,704,812	-613	-10,464,490	-23,188	-252,085	-85	-339,176	-1,283
SMC++	-7,710,701	-6,503	-10,448,611	-7,310	-252,185	-185	-338,244	-351
MSMC 2-Hap	-7,715,713	-11,514	-10,462,984	-21,682	-252,314	-314	-338,312	-418
MSMC 8-Hap	-7,711,087	-6,888	-10,518,421	-77,119	-252,350	-351	-341,278	-3,385
MSMC 4-Hap	-7,759,274	-55,075	-10,783,438	-342,137	-253,597	-1,598	-347,169	-9,275

	YRI: Figure 5C							
	Observed SFS ("Data"): 1000 Genomes, Whole Genome				Observed SFS ("Data"): 1000 Genomes, Neutral			
	SINGLETONS EXCLUDED		SINGLETONS INCLUDED		SINGLETONS EXCLUDED		SINGLETONS INCLUDED	
**Model**	**LL**	**Δ LL**	**LL**	**Δ LL**	**LL**	**Δ LL**	**LL**	**Δ LL**
Data to Data	-10,729,261	0	-15,946,971	0	-345,387	0	-507,708	0
Gutenkunst	-10,730,013	-752	-15,949,218	-2,247	-345,497	-110	-508,105	-397
SMC++	-10,744,311	-15,050	-15,991,411	-44,440	-345,857	-470	-508,588	-880
MSMC 2-Hap	-10,762,873	-33,612	-16,085,700	-138,729	-346,435	-1,047	-510,990	-3,282
MSMC 8-Hap	-10,874,066	-144,804	-16,683,783	-736,812	-349,917	-4,529	-528,106	-20,398
MSMC 4-Hap	-11,081,169	-351,908	-17,332,436	-1,385,465	-356,541	-11,154	-547,635	-39,927

[a]Denotes the best log-likelihood possible when replacing the proportions predicted by the model with the observed proportions from the SFS used by Gutenkunst *et al.* (2009) in their inference (see **Supplementary Note 4**).
[b]Denotes the model inferred by Gutenkunst *et al.* (2009) fit to the observed SFS
[c]Denotes the model inferred by Terhorst *et al.* (2017) using SMC++, a combined approach
[d]Denotes the demographic models inferred by Schiffels and Durbin (2014) using MSMC on 2, 4 and 8 haplotypes and scaled using their preferred mutation rate of 1.25×10^{-8} mutations/bp/gen
[e]Multinomial log-likelihood
[f]Δ LL = Model LL – Data LL; the table is sorted by the Δ LL value relative to the whole genome SFS without singletons (first two columns).

Table S5. Multinomial log-likelihoods comparing the fit of various models to the observed SFS from Gutenkunst *et al.* (2009), after trimming off ancient events and adjusting the ancestral size of the MSMC CEU 2-Haplotype model.

CEU						
**Model**	**Trimmed (Y/N)**[d]	$\mathbf{N_A}$	$\mathbf{N_A}$ **Notes**	**Multinomial LL**	**Δ LL (Model - Data)**	**Figure**
Data to Data[a]	**---**	**---**	**---**	**-21546**	0	3, S10, S12
Gutenkunst[b]	N	7,310	Gutenkunst N_A	-21555	-9	1, 3, S10, S12
MSMC 2-Hap[c]	Y	12,300	Gutenkunst N_{AF}[f]	-21584	-38	S12D
MSMC 2-Hap	Y	10,000	Commonly used N_A	-21587	-41	S12E
MSMC 2-Hap	Y	7,300	Gutenkunst N_A	-21653	-107	S12F
MSMC 2-Hap	N	7,300	Gutenkunst N_A	-21654	-109	S10F
MSMC 2-Hap	N	10,000	Commonly used N_A	-21656	-110	S10E
MSMC 2-Hap	N	12,300	Gutenkunst N_{AF}	-21658	-112	S10D
MSMC 2-Hap	N	19,834	Harm. mean of MSMC model prior to trim interval	-21665	-119	S10C
MSMC 2-Hap	N	23,261	MSMC *N* at trim interval	-21669	-123	S10B
MSMC 2-Hap	Y	19,834	Harm. mean of MSMC model prior to trim interval	-21681	-135	S12C
**MSMC 2-Hap**[e]	**N**	**41,261**	**MSMC N_A (oldest *N*) (unaltered model)**	**-21698**	**-152**	**1, 3, S10A**
MSMC 2-Hap	Y	23,261	MSMC *N* at trim interval	-21739	-193	S12B
MSMC 2-Hap	Y	41,261	MSMC N_A (oldest *N*)	-22001	-455	S12A

[a]Denotes the best log-likelihood possible when replacing the proportions predicted by the model with the observed proportions (see **Supplementary Note 4**).
[b]Denotes the model inferred by Gutenkunst *et al.* (2009) fit to the observed SFS
[c]Denotes versions of the CEU 2-Haplotype demographic model inferred by Schiffels and Durbin (2014) using MSMC and scaled using a mutation rate of 1.25×10^{-8} mutations/bp/gen. This model was adjusted to be trimmed to 225ky and/or have alternate ancestral sizes (N_A).
[d] "Trimmed" denotes MSMC models that were (Y) or were not (N) trimmed to remove events more ancient that 225.5kya.
[e]In bold is the original MSMC output, with no modifications to the model.
[f]The population expansion size inferred by Gutenkunst *et al.* (2009) prior to the CHB+CEU/YRI split.

Table S6. SNP Count Poisson likelihoods compared to the observed SFS from Gutenkunst *et al.* (2009), after trimming off ancient events and adjusting the ancestral size of the MSMC CEU 2-Haplotype model.

CEU								
**Model**	**Trim Y/N[d]**	**N_A**	**μ^f**	**N_A Notes**	**θ^g**	**Poisson LL**	**Δ LL (Model - Data)**	**Figure**
Data to Data[a]	------	------	----	------	------	**44688**	0	4, S11, S13
Gutenkunst[b]	N	7,310	2.35	Gutenkunst N_A	2776	44679	-9	1, 4, S11, S13
MSMC 2-Hap[c]	N	7,300	1.25	Gutenkunst N_A	1475	44569	-119	S11F
MSMC 2-Hap	N	10,000	1.25	Commonly used N_A	2020	44563	-125	S11E
MSMC 2-Hap	N	12,300	1.25	Gutenkunst N_Af[h]	2485	44557	-131	S11D
MSMC 2-Hap	N	19,834	1.25	Harm. mean of MSMC model prior to trim interval	4006	44530	-158	S11C
MSMC 2-Hap	N	23,261	1.25	MSMC N at trim interval	4699	44515	-173	S11B
MSMC 2-Hap	Y	19,834	1.25	Harm. mean of MSMC model prior to trim interval	4006	44501	-187	S13C
MSMC 2-Hap	Y	12,300	1.25	Gutenkunst N_Af	2485	44472	-216	S13D
**MSMC 2-Hap[e]**	**N**	**41,261**	**1.25**	**MSMC N_A (oldest N)**	**8335**	**44410**	**-278**	**1, 4, S11A**
MSMC 2-Hap	Y	23,261	1.25	MSMC N at trim interval	4699	44263	-425	S13B
MSMC 2-Hap	Y	10,000	1.25	Commonly used N_A	2020	44221	-467	S13E
MSMC 2-Hap	Y	7,300	1.25	Gutenkunst N_A	1475	43674	-1014	S13F
MSMC 2-Hap	Y	41,261	1.25	MSMC N_A (oldest N)	8335	41724	-2964	S13A

[a]Denotes the best log-likelihood possible when replacing the counts predicted by the model with the observed counts (see **Supplementary Note 4**).
[b]Denotes the model inferred by Gutenkunst *et al.* (2009) fit to the observed SFS and using a mutation rate of 2.35×10^{-8} mutations/bp/gen.
[c]Denotes versions of the CEU 2-Haplotype demographic model inferred by Schiffels and Durbin (2014) using MSMC and scaled using a mutation rate of 1.25×10^{-8} mutations/bp/gen. This model was adjusted to be trimmed to 225ky and have alternate ancestral sizes.
[d] "Trimmed" denotes MSMC models that were (Y) or were not (N) trimmed to remove events more ancient that 225.5kya.
[e]In bold is the original MSMC output, with no modifications to the model.
[f]Mutation rate used for scaling models and calculating θ (units of 10^{-8} mutations per base per generation)
[g]$\theta = 4 * N_A * \mu *$ sequence length (4.04Mb)
[h]The population expansion size inferred by Gutenkunst *et al.* (2009) prior to the CHB+CEU/YRI split

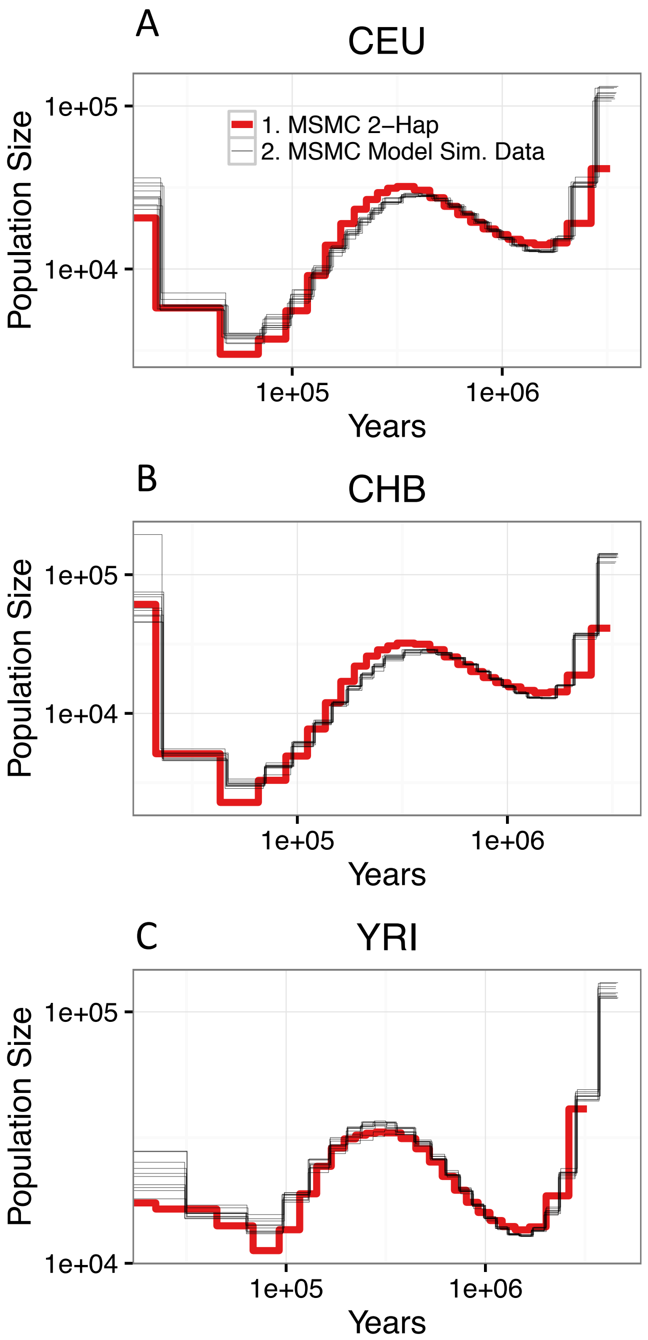

Figure S1. MSMC 2-Haplotype can recapitulate its own trajectories from simulated data. 10 diploid 2.4Gb ‘genomes’ were simulated under the MSMC 2-Haplotype inferred demographic model (heavy red line; Schiffels and Durbin (2014)) for the CEU (**A**), CHB (**B**) and YRI (**C**) populations. MSMC 2-Haplotype was then run on the simulated data to see how closely the program is able to recapitulate this particular demography (fine black lines), and to validate our method of converting MSMC trajectories into stepwise demographic models.

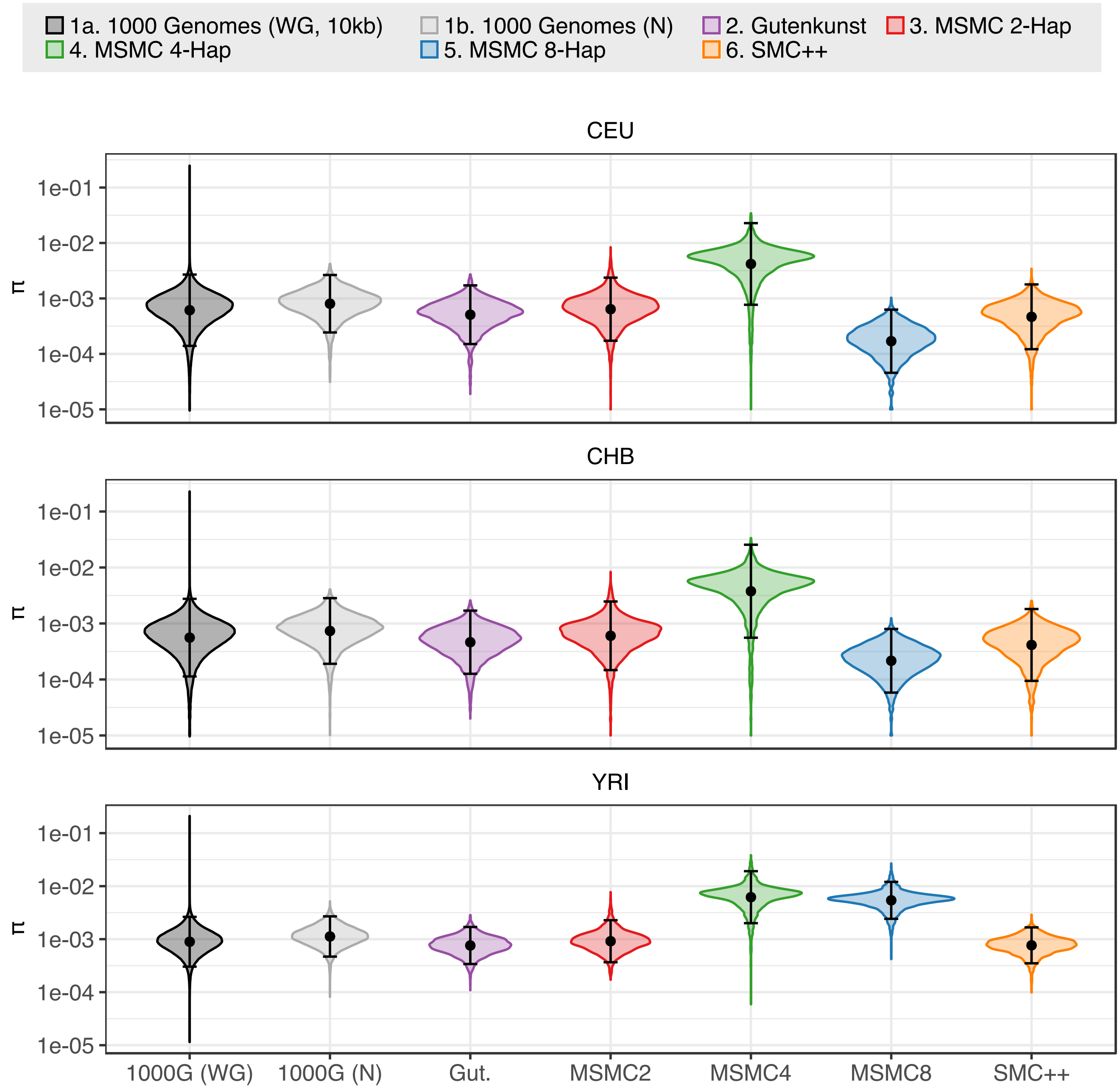

Figure S2. Kernel density distribution of expected heterozygosity (π per site). Heterozygosity was calculated across 10kb windows from whole genome 1000 Genomes project data for CEU (A), CHB (B), and YRI (C), from 6333 x 10kb putatively neutral windows from 1000 Genomes, and 6300 x 10kb blocks for data simulated under each demographic model. The black dot and bars indicate the mean ± two standard deviations for each distribution. Note the log-10 scaling on the y-axis.

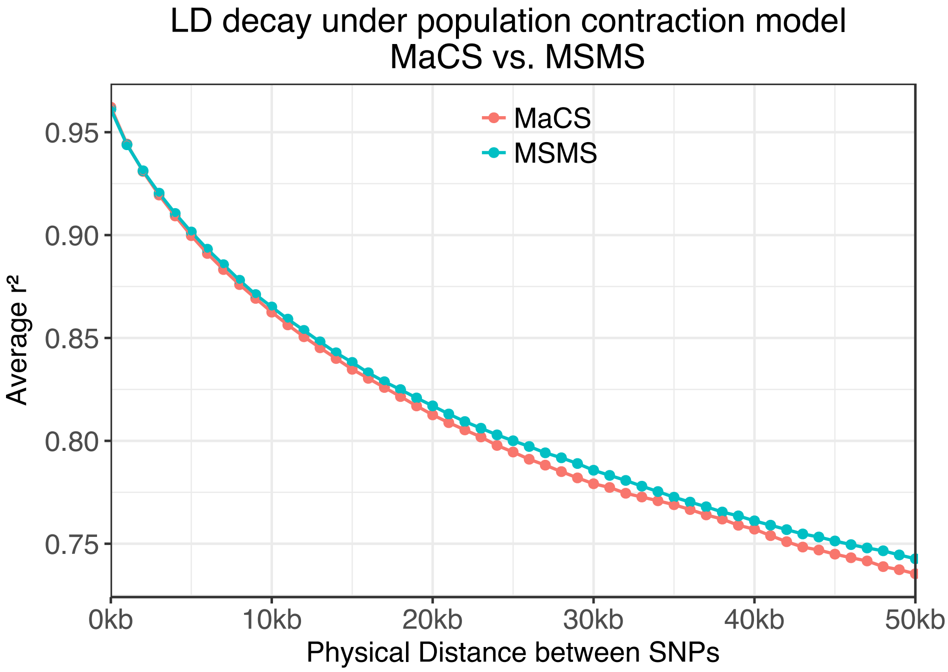

Figure S3. **Use of the SMC' approximation does not affect LD pattern of simulated data.** Data was simulated data as described in **Methods** under a decline model (population going from 100,000 to 1,000 individuals) using both MaCS (Chen *et al.* 2009) which uses the SMC approximation) and MSMS (Ewing and Hermisson 2010) which does not. We found that both simulators produced highly similar LD decay curves, indicating that it is not the SMC approximation that is causing the LD decay curves under the published demographic models not to match the 1000 Genomes data.

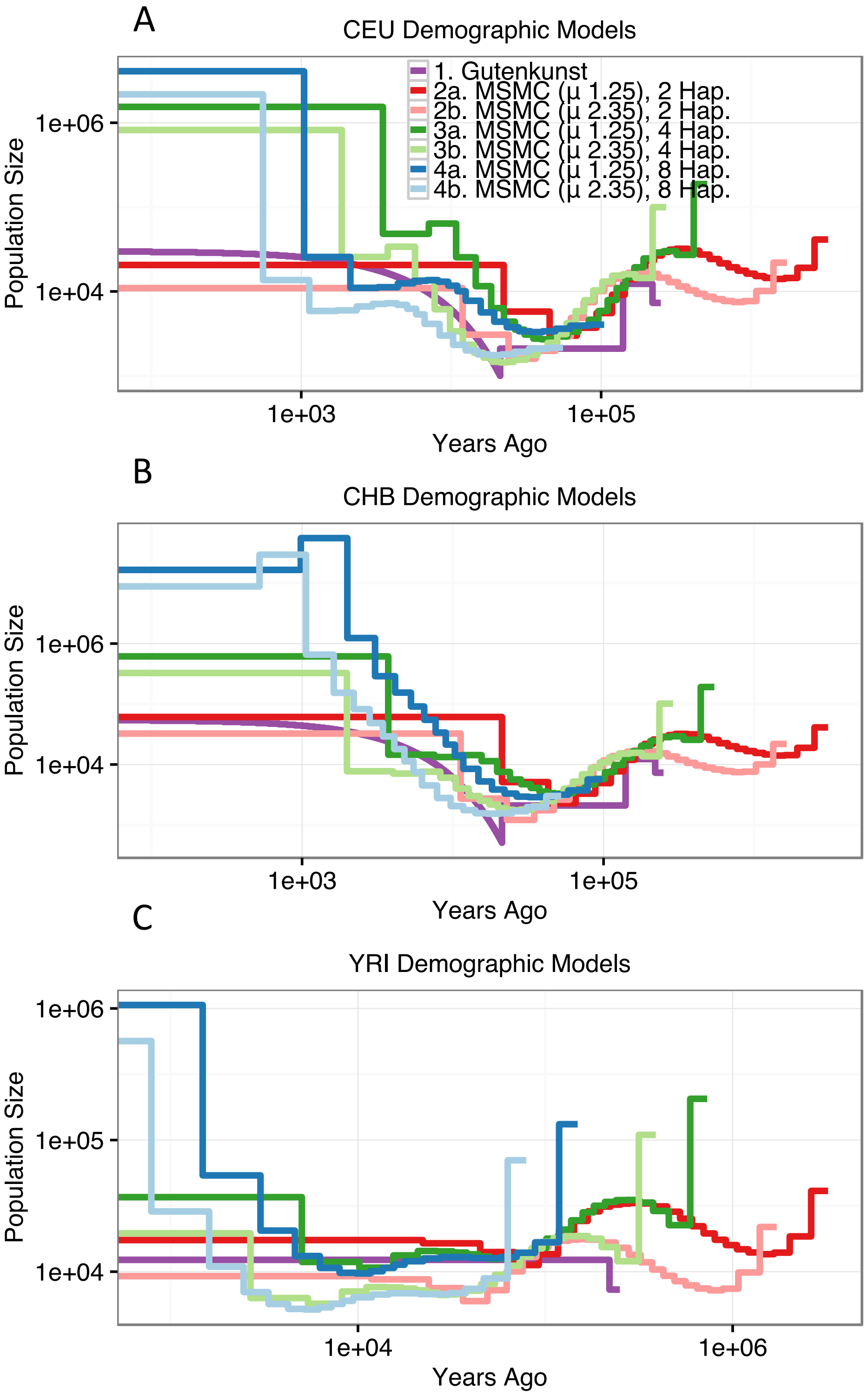

Figure S4. Demographic histories for the CEU (A), CHB (B), and YRI (C) populations. Trajectories are log-scaled and in terms of physical units (diploid individuals and years). Models were either inferred using SFS-based methods (“Gutenkunst”) by Gutenkunst *et al.* (2009) or from a sequentially Markovian coalescent-based approach (“MSMC”) from two, four and eight haplotypes by Schiffels and Durbin (2014). Lighter colors represent rescaling of the Schiffels and Durbin (2014) model using a mutation rate of 2.35x10^{-8} mutations/bp/gen (as opposed to 1.25x10-8 mutations/bp/gen as presented in Schiffels and Durbin (2014)), approximately halving population sizes and times of size changes (**Supplementary Note 5**).

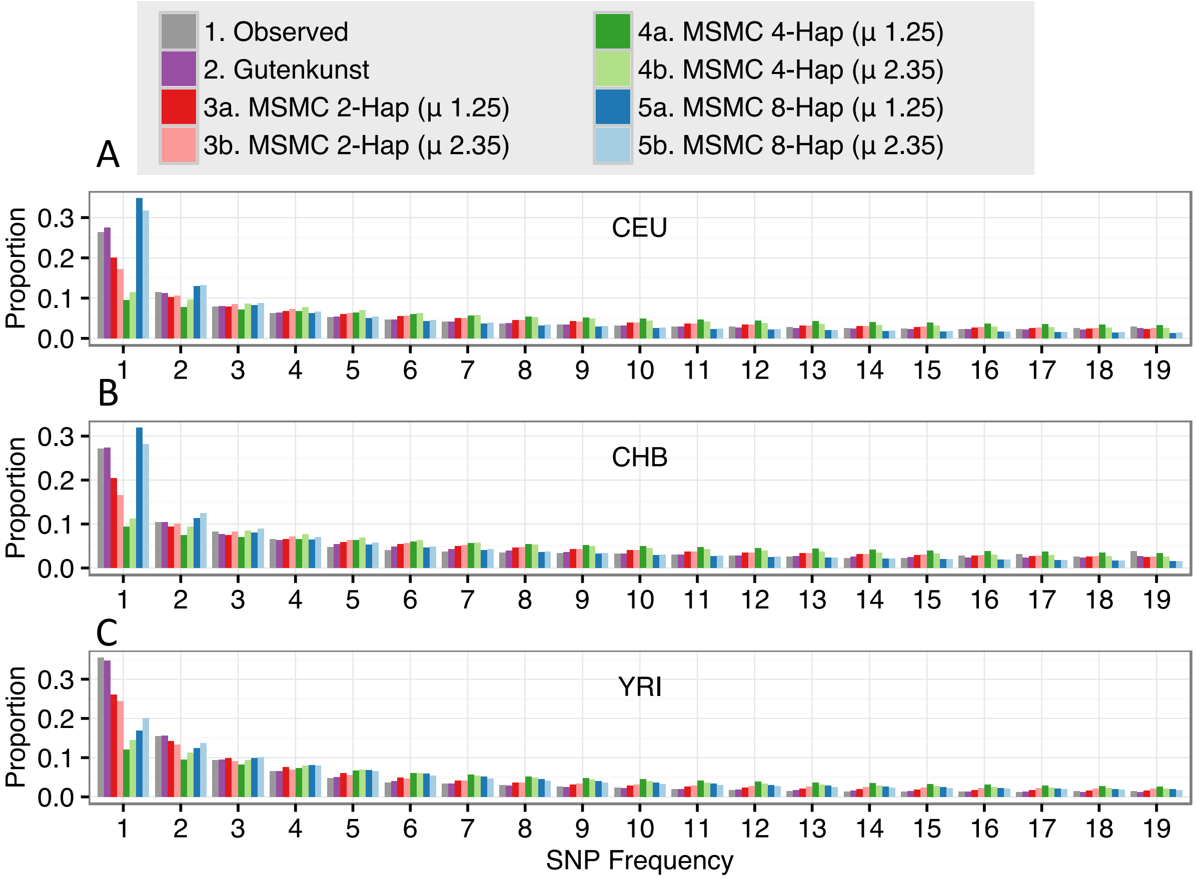

Figure S5. Proportional site frequency spectra for CEU (A), CHB (B), and YRI (C) populations with MSMC models scaled using an alternate mutation rate. The "Observed" SFS is from noncoding sequence used by Gutenkunst *et al.* (2009) to infer demographic histories for these three populations. The "Gutenkunst" SFS is the predicted SFS under the models inferred in Gutenkunst et al (2009). The "MSMC" SFSs are those expected under the demographic histories inferred using MSMC from two, four and eight haplotypes by Schiffels and Durbin (2014). The MSMC estimates were scaled using either a mutation rate of 1.25 $\times 10^{-8}$ mutations/bp/gen ("µ 1.25") as in Schiffels and Durbin (2014), or 2.35$\times 10^{-8}$ mutations/bp/gen as in Gutenkunst *et al.* (2009) ("µ 2.35") (**Supplementary Note 5; Figure S3)**. Note that none of the MSMC SFSs appear to match the Observed SFS (gray), regardless of mutation rate used.

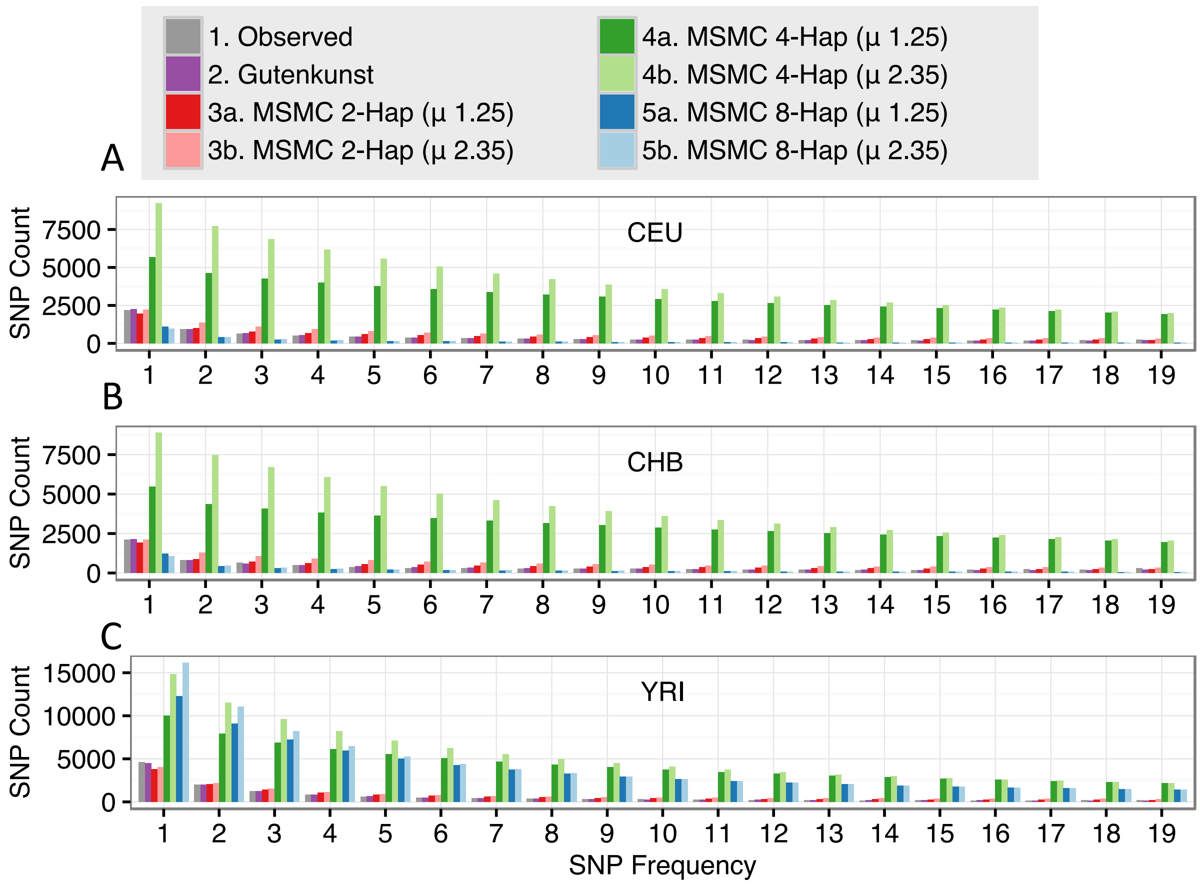

Figure S6. SNP count site frequency spectra using the counts of SNPs for the CEU (A), CHB (B), and YRI (C) populations with MSMC models scaled using an alternate mutation rate. The "Observed" SFS is from noncoding sequence used by Gutenkunst *et al.* (2009) to infer demographic histories for these three populations. The "Gutenkunst" SFS is the predicted SFS under the models inferred in Gutenkunst et al (2009). The "MSMC" SFSs are those expected under the demographic histories inferred using MSMC from two, four and eight haplotypes by Schiffels and Durbin (2014). The MSMC estimates were scaled using either a mutation rate of 1.25 x10^{-8} mutations/bp/gen ("µ 1.25") as in Schiffels and Durbin (2014), or 2.35x10^{-8} mutations/bp/gen as in Gutenkunst *et al.* (2009) ("µ 2.35") (**Supplementary Note 5; Figure S3**). SFSs are scaled using the ancestral population size given by each model, the mutation rate used to scale each version of the model, and the sequence length of the empirical dataset (4.04Mb).

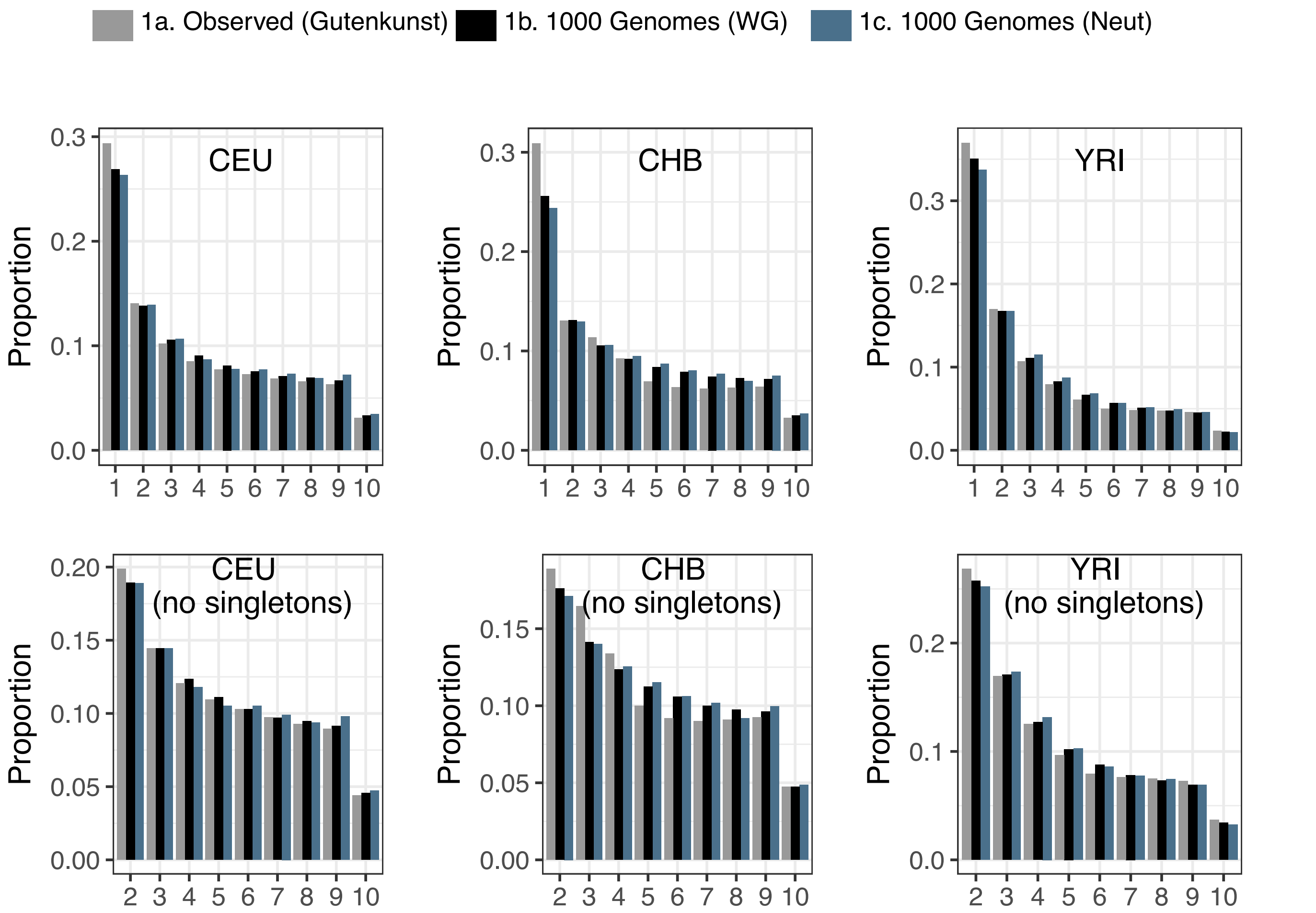

Figure S7. Comparison of the three empirical folded proportional SFSs. 1a. Observed (Gutenkunst) is based on intronic sequence and was used by Gutenkunst *et al.* (2009) to infer demographic histories for CEU, CHB and YRI. 1b. 1000 Genomes (WG) is based on low-coverage high-throughput sequencing data from the 1000 Genomes Project. 1c. 1000 Genomes (Neutral) is based on putatively neutral regions in the same 1000 Genomes dataset. SFSs are shown with singletons (top row), and renormalized without singletons, as the singletons category in low-coverage datasets can be unreliable (bottom row).

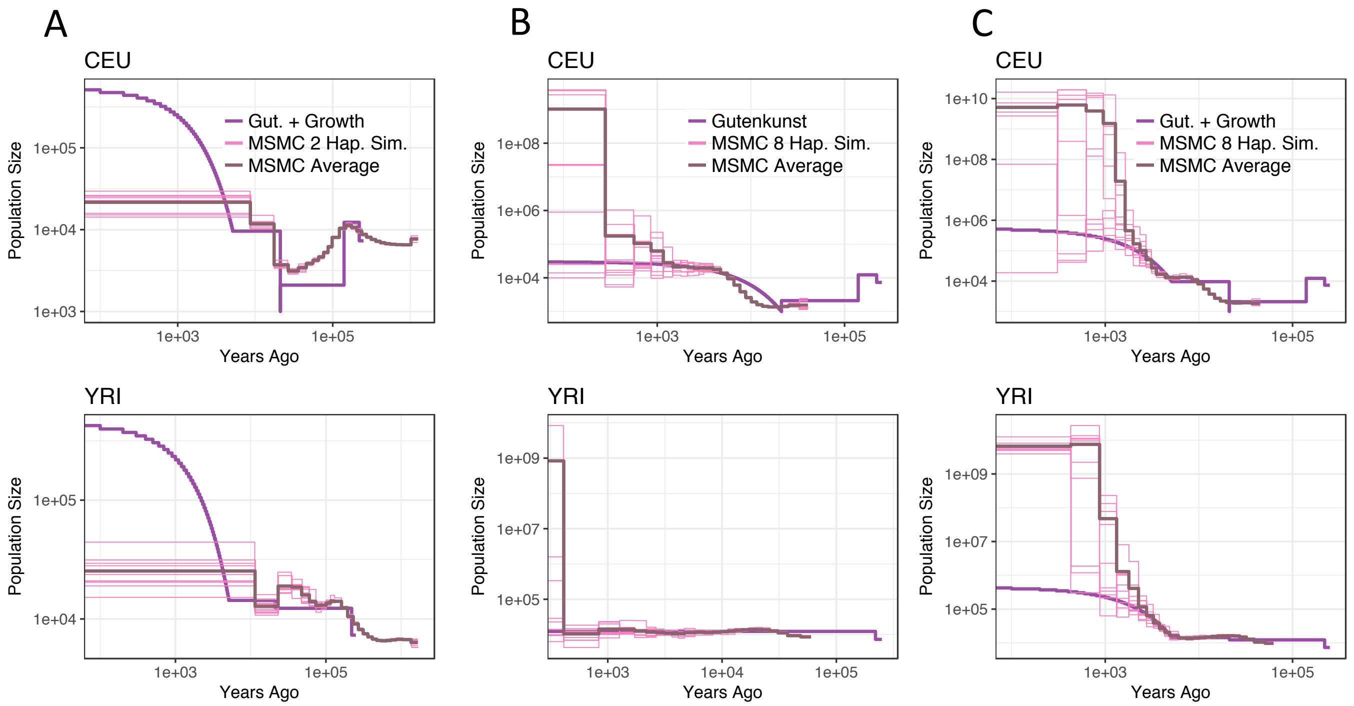

Figure S8. MSMC 8-Haplotype overestimates explosive recent growth, while MSMC 2-Haplotype underestimates it. For the CEU and YRI populations, we simulated 10 genomic 2-haplotype and 8-haplotype datasets under the Gutenkunst model ("Gutenkunst"), and a modified version of the Gutenkunst model that incorporates explosive recent growth inferred by Tennessen *et al.* (2012) ("Gut. + Growth"). MSMC was then used to infer the demographic histories from these simulated datasets. The demographic histories underlying our simulations are shown by the think purple lines, and the results of (**A**) MSMC 2-Haplotype ("MSMC 2 Hap. Sim.") and (**B-C**) MSMC 8-Haplotype ("MSMC 8 Hap. Sim.") (with the --fixedRecombination option) run on these data are represented by the fine pink lines. The average of the MSMC trajectories is noted with the thick dark pink line. MSMC 2-Haplotype run on data simulated under the original Gutenkunst model can be seen in main text **Figure 7A**. While MSMC 2-Haplotype relatively accurately estimates the moderate recent growth in the Gutenkunst model (**Figure 7**), it vastly underestimates explosive recent growth in the Gut. + Growth model (**A**), a known weakness of the method. However, MSMC 8-Haplotype, which is thought to be better at estimating recent growth, vastly overestimates growth for both models (**B, C**), particularly in the case of (**C**) recent explosive growth ("Gut. + Growth").

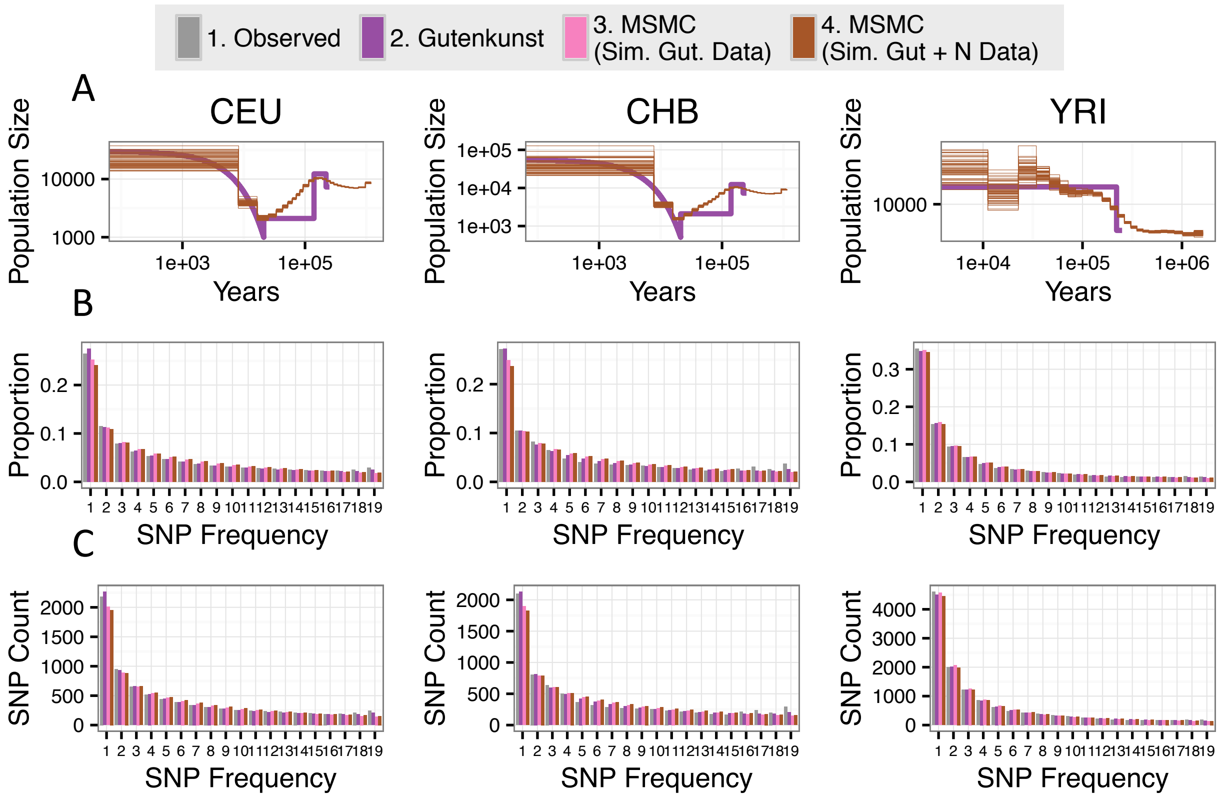

Figure S9. Adding Neanderthal admixture to the Gutenkunst *et al.* (2009) model does not affect MSMC inference. (**A**) shows the results of running MSMC 2-Haplotype on 50 independent 2-haplotype datasets simulated under the Gutenkunst *et al.* (2009) model of human demographic history with the addition of 2% Neanderthal admixture 2000 generations ago ("Gutenkunst," heavy purple line). The resulting MSMC 2-Haplotype trajectories ("MSMC Sim. Gut. + N Data," fine brown lines) show the MSMC trajectories inferred from these 50 datasets. Note that these trajectories accurately track the demographic model used to simulate the data, and do not dramatically differ from the trajectories in Figure 4. (**B**) and (**C**) show proportional and SNP count site frequency spectra for each population, respectively. The gray bars (Observed) denote the Observed SFS used by Gutenkunst *et al.* (2009). The purple bars denote the expected SFS under the inferred Gutenkunst demographic models. The pink bars denote the expected SFS under the average of the 50 MSMC demographic model trajectories for each population from data simulated under the Gutenkunst model, as in **Figure S8**. The brown bars denote the expected SFS under the average of the 50 MSMC demographic model trajectories for each population from data simulated under the Gutenkunst mode plus Neanderthal admixture. Note that the addition of Neanderthal admixture does not disrupt the MSMC trajectories (brown), or make the SFS based on those trajectories (brown bars) differ from the other SFSs.

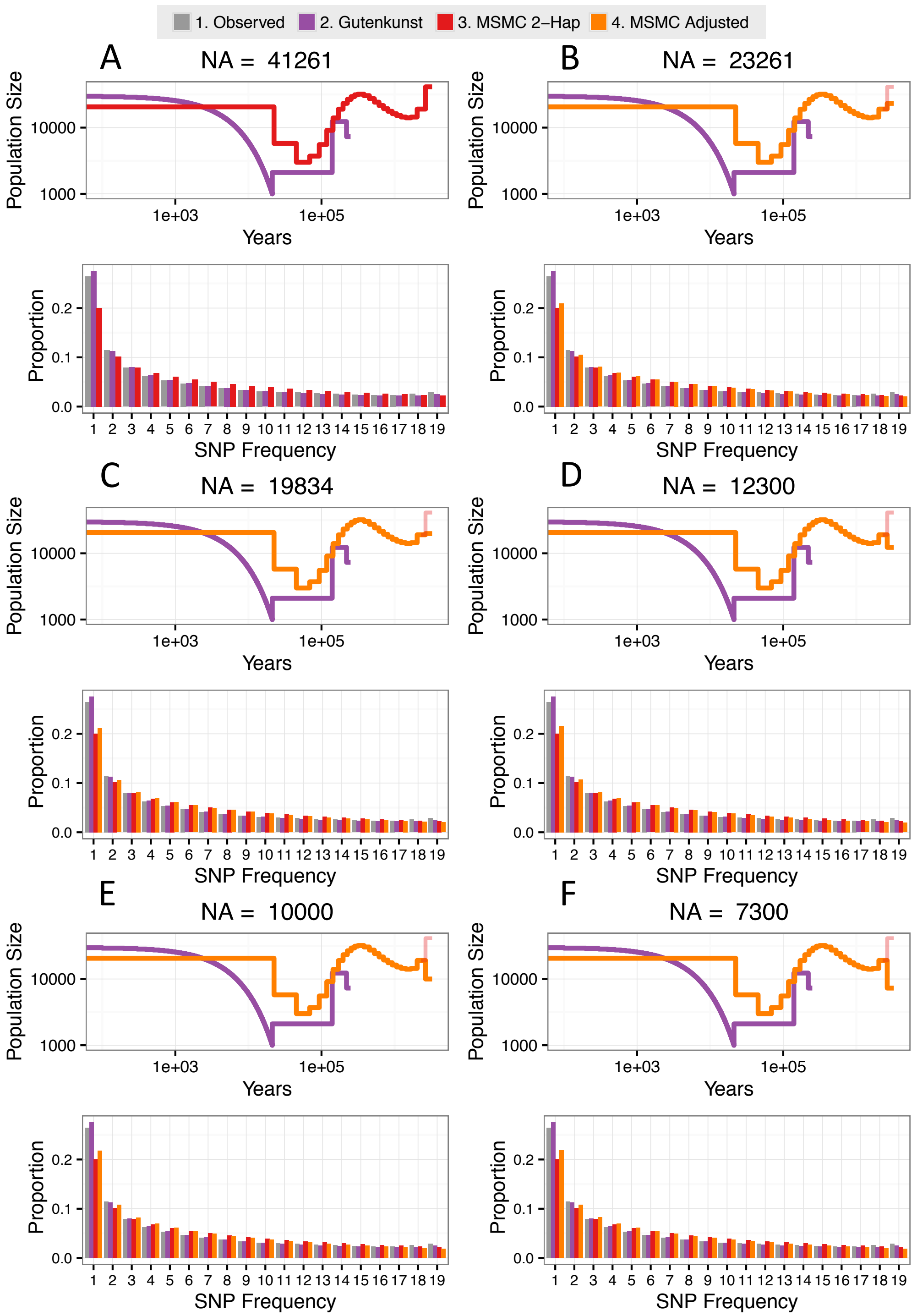

Figure S10. Adjusting the ancestral size does not result in a better fit of the proportional SFS expected under the MSMC CEU 2-Haplotype trajectory to the observed proportional SFS. Adjustments were made to the MSMC CEU 2-Haplotype demographic model (Schiffels and Durbin 2014) so that it has a range of different ancestral sizes (N_A) (**A-F**, see **Supplementary Note 7**). The upper plot in each pair shows the adjusted demographic model used to generate the expected SFS (orange); the faded red line shows the unaltered original model. Below each trajectory are the proportional SFSs for that scenario. The "Observed" SFS was used by Gutenkunst *et al.* (2009) to infer demographic histories for these three populations. The "Gutenkunst" SFS is the expected SFS under the models they inferred in that study. The "MSMC 2-Hap" SFS is the SFS expected under the original unadjusted model, while the "MSMC Adjusted" SFS is expected under the adjusted model. Log-likelihoods can be found in **Table S5**. Note that the adjustments here do not result in a substantial improvement in fit to the observed SFS.

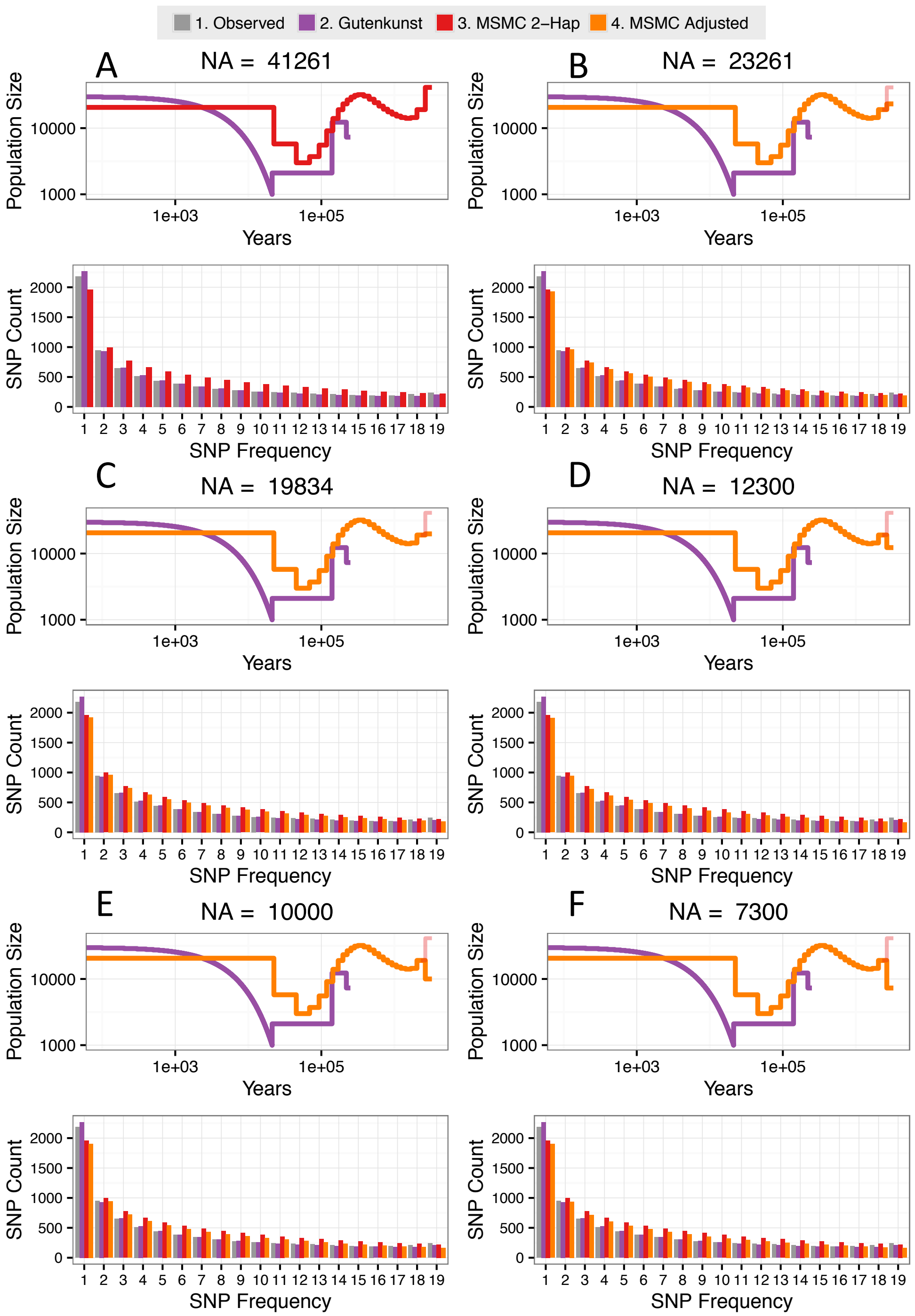

Figure S11. Adjusting the ancestral size results in a slightly better fit of the SNP count SFS expected under the MSMC CEU 2-Haplotype trajectory to the observed SNP count SFS. Adjustments were made to the MSMC CEU 2-Haplotype demographic model (Schiffels and Durbin 2014) so that it has a range of different ancestral sizes (N_A) (**A-F**, see **Supplementary Note 7**). The upper plot in each pair shows the adjusted demographic model used to generate the expected SFS (orange); the faded red line shows the unaltered original model. Below each trajectory are the SNP Count SFSs for that scenario, scaled up using the ancestral size for each model, the mutation rate used in each model ($1.25\text{x}10^{-8}$ mutations/bp/gen for the MSMC models, $2.35\text{x}10^{-8}$ mutations/bp/gen for the Gutenkunst model) and the length of the empirical dataset (4.04Mb). The "Observed" SFS was used by Gutenkunst *et al.* (2009) to infer demographic histories for these three populations. The "Gutenkunst" SFS is the expected SFS under the models they inferred in that study. The "MSMC 2-Hap" shows the SFS expected under the unadjusted model, while the "MSMC Adjusted" SFS is expected under the adjusted model. Log-likelihoods can be found in **Table S6**.

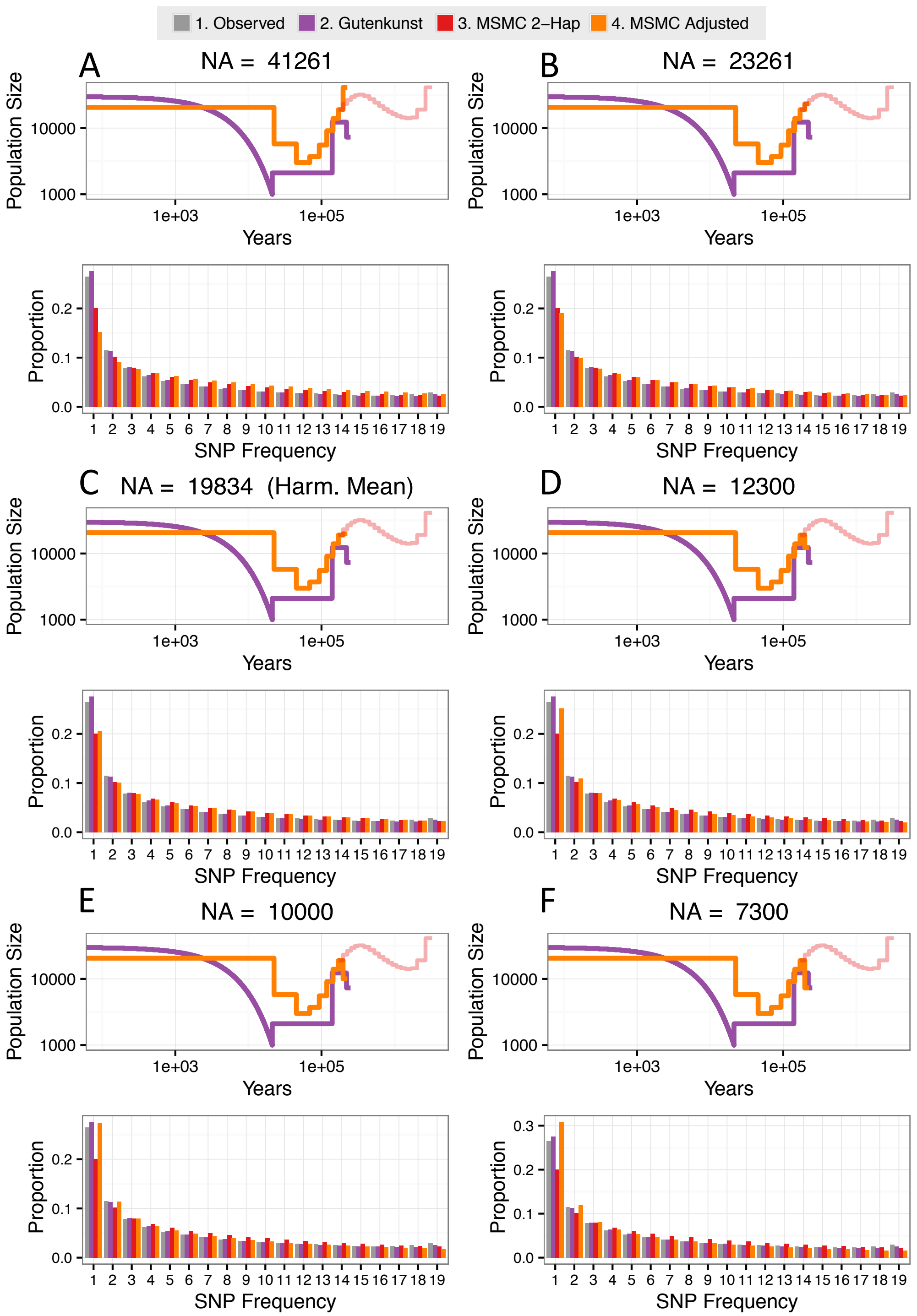

Figure S12. Trimming away the portion of the trajectory >225kya and changing the ancestral size (N_A) improved the fit of the proportional SFS expected under the MSMC CEU 2-Haplotype trajectory results to the observed proportional SFS. Adjustments were made to the MSMC CEU 2-Haplotype demographic model (Schiffels and Durbin 2014) so that events older than 225kya are removed and it has a range of different ancestral sizes (N_A) (**A-F**, see **Supplementary Note 7**). The upper plot in each pair shows the adjusted demographic model used to generate the expected SFS (orange); the faded red line shows the unaltered original model. Below each trajectory are the proportional SFSs for that scenario. The "Observed" SFS was used by Gutenkunst *et al.* (2009) to infer demographic histories for these three populations. The "Gutenkunst" SFS is the expected SFS under the models they inferred in that study. The "MSMC 2-Hap" shows the SFS expected under the unadjusted model, while the "MSMC Adjusted" SFS is expected under the adjusted model. Log-likelihoods can be found in **Table S5**.

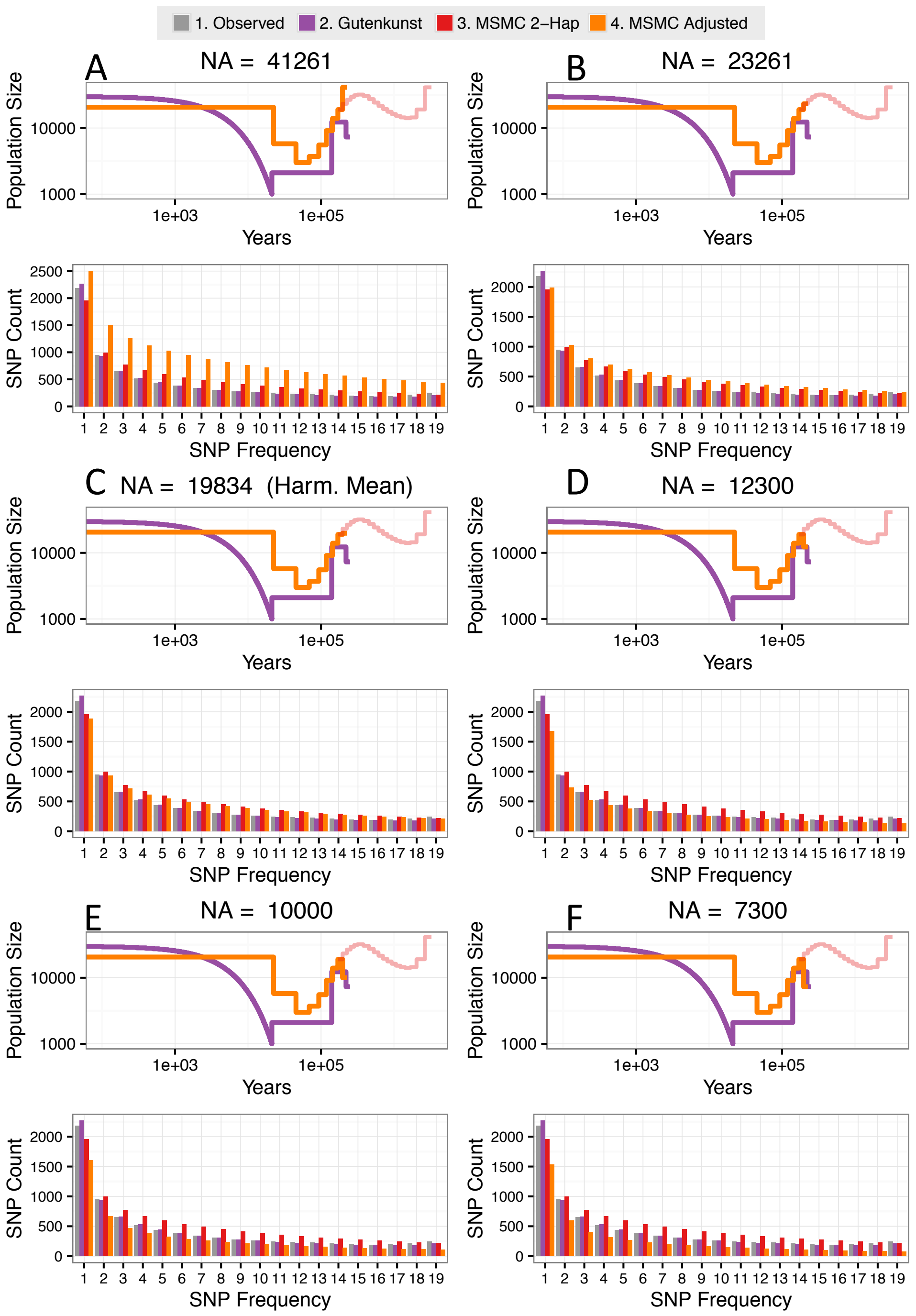

Figure S13. Trimming away the portion of the trajectory >225kya and changing the ancestral size (N_A) did not improve the fit of the SNP count SFS expected under the MSMC CEU 2-Haplotype trajectory results to the observed SNP count SFS. Adjustments were made to the MSMC CEU 2-Haplotype demographic model (Schiffels and Durbin 2014) so that events older than 225kya are removed and it has a range of different ancestral sizes (N_A) (**A-F**, see **Supplementary Note 7**). The upper plot in each pair shows the adjusted demographic model used to generate the expected SFS (orange); the faded red line shows the unaltered original model. Below each trajectory are the SNP Count SFSs for that scenario, scaled up using the ancestral size for each model, the mutation rate used in each model (1.25×10^{-8} mutations/bp/gen for the MSMC models, 2.35×10^{-8} mutations/bp/gen for the Gutenkunst model) and the length of the empirical dataset (4.04Mb). The "Observed" SFS was used by Gutenkunst *et al.* (2009) to infer demographic histories for these three populations. The "Gutenkunst" SFS is the expected SFS under the models they inferred in that study. The "MSMC 2-Hap" shows the SFS expected under the unadjusted model, while the "MSMC Adjusted" SFS is expected under the adjusted model. Log-likelihoods can be found in **Table S6.**

SOURCES CITED IN SUPPLEMENTARY INFORMATION.

Adams, A. M., and R. R. Hudson, 2004 Maximum-likelihood estimation of demographic parameters using the frequency spectrum of unlinked single-nucleotide polymorphisms. Genetics 168: 1699–1712.

Arbiza, L., E. Zhong, and A. Keinan, 2012 NRE: a tool for exploring neutral loci in the human genome. BMC Bioinformatics 13: 301.

Chen, G. K., P. Marjoram, and J. D. Wall, 2009 Fast and flexible simulation of DNA sequence data. Genome Res 19: 136–142.

Drmanac, R., A. B. Sparks, M. J. Callow, A. L. Halpern, N. L. Burns *et al.*, 2010 Human genome sequencing using unchained base reads on self-assembling DNA nanoarrays. Science 327: 78–81.

Ewing, G., and J. Hermisson, 2010 MSMS: a coalescent simulation program including recombination, demographic structure and selection at a single locus. Bioinformatics 26: 2064–2065.

Gutenkunst, R. N., R. D. Hernandez, S. H. Williamson, and C. D. Bustamante, 2009 Inferring the joint demographic history of multiple populations from multidimensional SNP frequency data. PLoS Genet 5: e1000695.

Harris, K., and R. Nielsen, 2016 The genetic cost of Neanderthal introgression. Genetics 203: 881–891.

Lohmueller, K. E., C. D. Bustamante, and A. G. Clark, 2010 The effect of recent admixture on inference of ancient human population history. Genetics 185: 611–622.

Schiffels, S., and R. Durbin, 2014 Inferring human population size and separation history from multiple genome sequences. Nat Genet 46: 919–925.

Takahata, N., Y. Satta, and J. Klein, 1995 Divergence time and population size in the lineage leading to modern humans. Theor Popul Biol 48: 198–221.

Tennessen, J. A., A. W. Bigham, T. D. O'Connor, W. Fu, E. E. Kenny *et al.*, 2012 Evolution and functional impact of rare coding variation from deep sequencing of human exomes. Science 337: 64–69.

Terhorst, J., J. A. Kamm, and Y. S. Song, 2017 Robust and scalable inference of population history from hundreds of unphased whole genomes. Nat Genet 49: 303–309.

Wakeley, J., 2009 *Coalescent theory: an introduction*. Roberts & Co. Publishers., Greenwood Village.
